# Hospitals in the United States

**Published:** 1905-10-28

**Authors:** 


### HOSPITAL ADMINISTRATION.

### CONSTRUCTION AND ECONOMICS.

### HOSPITALS IN THE UNITED STATES.

### THE BOSTON CITY HOSPITAL.

By our Special Commissioner.

This hospital was opened forty-one years ago, and for twenty-eight years Dr. George H. M. Rowe has been the superintendent and resident physician. During his term of office many material changes in the administration and structure of the hospital have taken place, and it is now one of the best organised and most efficient city hospitals in the United States.

#### Control Expenditure.

The history of the Boston City Hospital, in common with many others of the same type, illustrates the absence of any necessity for hospital managers anywhere to incur large deficits each year. A deficit in fact can be permitted or prevented at will. The system of finance at this hospital is simple. Each year the trustees and managers submit a budget and estimates to the comptroller of the city of Boston, setting forth the actual money which the city is asked to appropriate for the current year, in order to meet the whole of the expenditure. For the year ending January, 1905, the city appropriation amounted to $485,000, or, say, £100,000 in round figures. To this must be added $2,000 derived from interest, trust funds, etc., and the expenditure was so regulated as to be just within the appropriation. The hospital and its departments contain altogether 935 beds, of which 20 are located in the Relief Station, in a densely populated portion of Boston, and 36 are placed in the Convalescents' Home. It will be seen, therefore, that this hospital approaches the maximum accommodation (1,000 beds), which the highest authorities hold to be desirable or possible in an institution devoted to the care of patients suffering from acute diseases.

It further appears, that owing to the failure of the contractors whereby the completion of the out-patients' department was delayed, the trustees cut down the appropriation asked for for the current year, so that when the new contractors handed over the building earlier than it was expected a deficit would have occurred at the end of the fiscal year had the trustees at once opened these new buildings for the reception of patients. But as the appropriation made by the city of Boston was only adequate to defray the cost of occupancy for the parts of the hospital then in commission, it was decided not to open these new buildings until the commencement of the ensuing financial year, when an adequate appropriation was asked for and obtained from the city comptroller. This brief statement of the working of a great city hospital in the United States should be fruitful in instruction to the managers of every voluntary hospital dependent upon endowments and free gifts for its maintenance; for it proves to demonstration that it is within the power of the managers of every hospital to so regulate its expenditure, and the amount of work done each year, as to insure that no deficit shall occur.

#### Abolish Deficits.

We are strongly of opinion that, if every voluntary hospital were to adopt the system of budgets and estimates, and to submit a statement to the public at the commencement of each year, showing that only so much work could be undertaken unless the public subscribed more money than they had heretofore done, when the full working capacity of the hospital would be maintained, all the funds that such a hospital might require to meet the needs of the actual poor and suffering would be forthcoming. Spurgeon, who was one of the most successful beggars in his day, never permitted any indebtedness to be incurred unless he had money in hand to defray it. There is no less popular appeal than an appeal to wipe out a deficit, a fact which we commend to the earnest consideration of all who have to raise money for hospital purposes in the present time.

#### Superficial Area and Overcrowding Site.

The buildings of the Boston City Hospital represent several periods of hospital construction. When we made our inspection on the 4th of October it interested us a good deal to notice that the old surgical and medical pavilions were still efficient, though they contained several marked defects in regard to ventilation and other matters. A great drawback which this hospital labours from is that masses of buildings have been crowded upon too limited a site. In these days there is no justification for erecting a huge wing in such close contiguity to existing buildings that the hygienic efficiency of both old and new erections are permanently interfered with. This fault is glaringly present in regard to all the ward pavilions on the left-hand side of the main entrance. The buildings on the right-hand side were formerly free from this defect, but the recent erection of a surgical building to provide adequate operation theatres, with all essentials, and having the quarters of the resident medical staff in the upper stories, has interfered very gravely with the hygienic efficiency of the Miles Surgical Ward, a one-story wooden building originally put up as a temporary expedient, but which has proved so efficient—like many of its type—that it has continued to be occupied by surgical cases, and is still one of the most popular and attractive wards in connection with the hospital. We presume, however, having regard to the present building regulations of the city of Boston, that as this male surgical ward must be removed, owing to its juxtaposition to the new surgical building, that it will not be re-erected, as there does not appear to be any ground available for the purpose. We observed that practically the only open space left on the site—that behind the open ward building on the left-hand side of the entrance—had been staked out to show the space which a new pavilion block would take up. We cannot, however, credit that this small air space, so desirable—nay, so essential—to the wellbeing of the patients and the general health of the hospital, will ever be devoted to future extensions, though it is marked on the plan for that purpose.

### The Pathological Building.

One of the most efficient units connected with this hospital is the pathological building. The amount of clinical and scientific work which has been, and is being carried on in this building is worthy of the reputation of one of the greatest of medical schools. Everything appears to be conducted with the maximum of intelligence and thoroughness, and we were particularly struck with the arrangements made to preserve and classify pathological material in a form which enables it to be readily supplied for teaching purposes to other schools and hospitals as and when required. In connection with this block is the autopsy theatre, the mortuary, morgue, and mortuary chapel. All the arrangements connected with these necessary adjuncts to a large city hospital, where there are nearly a thousand deaths per annum, were excellent, and revealed an amount of care and thought which may make this new pathological building the basis for the work of many people who may be charged with the responsibility of designing new hospitals, and evolving an efficient pathological unit.

### Matron's Department and Nursing.

The new Nurses' Home, known as the "Voce House," presents some attractive features, the bedroom accommodation for individual nurses being excellent, as well as the hall and recreation-room space, which is so ample as to raise the question whether it may not be, in fact, excessive for the number of nurses accommodated. We were, however, particularly struck with one glaring defect, namely, the inadequate and miserable accommodation set apart as the quarters of the superintendent of nurses and matron. The matron is provided with an ample office of suitable proportions and appearance, and we can only put down the failure to provide adequate accommodation for her comfort during off-duty hours to an absence of appreciation on the part of the trustees of the claims which such an officer has on their consideration, and of the wisdom of providing that her private quarters are hygienic and in all respects worthy of a great institution like the Boston City Hospital. As the matter stands at present we do not think there is any other hospital of anything like the same size where the matron is so badly housed, and for this reason we hope that wiser counsels may prevail and that more suitable quarters for the matron may be provided without delay. One of the best features of the existing plans is the ground floor of the administration block, where the office accommodation and its arrangement present many advantages which are well worthy of imitation by modern hospital constructors. We congratulate Dr. Rowe and his staff upon the general efficiency which was everywhere apparent throughout the institution.

The new out-patients' department has been hampered by the restrictions of the site upon which it stands, but as we hope one day to give a plan of this department, we need not further refer to it upon the present occasion. This hospital has several notable features, including the medical library, the department for contagious diseases (with accommodation for 264 patients), the relief station in the centre of the town, and a large ambulance department. We have not space to give particulars of each of these. It is only right, however, that we should specially commend the ambulance buildings and their equipment, where everything is carried out in the most efficient way under the direction of a superintendent who was once a jockey in England. He keeps the horses, harness rooms, ambulances, and everything appertaining thereto, so smart as to make them, in fact, probably the smartest carriage vehicles in the city of Boston.

